# jMOTU and Taxonerator: Turning DNA Barcode Sequences into Annotated Operational Taxonomic Units

**DOI:** 10.1371/journal.pone.0019259

**Published:** 2011-04-25

**Authors:** Martin Jones, Anisah Ghoorah, Mark Blaxter

**Affiliations:** Institute of Evolutionary Biology, University of Edinburgh, Edinburgh, United Kingdom; British Columbia Centre for Excellence in HIV/AIDS, Canada

## Abstract

**Background:**

DNA barcoding and other DNA sequence-based techniques for investigating and estimating biodiversity require explicit methods for associating individual sequences with taxa, as it is at the taxon level that biodiversity is assessed. For many projects, the bioinformatic analyses required pose problems for laboratories whose prime expertise is not in bioinformatics. User-friendly tools are required for both clustering sequences into molecular operational taxonomic units (MOTU) and for associating these MOTU with known organismal taxonomies.

**Results:**

Here we present jMOTU, a Java program for the analysis of DNA barcode datasets that uses an explicit, determinate algorithm to define MOTU. We demonstrate its usefulness for both individual specimen-based Sanger sequencing surveys and bulk-environment metagenetic surveys using long-read next-generation sequencing data. jMOTU is driven through a graphical user interface, and can analyse tens of thousands of sequences in a short time on a desktop computer. A companion program, Taxonerator, that adds traditional taxonomic annotation to MOTU, is also presented. Clustering and taxonomic annotation data are stored in a relational database, and are thus amenable to subsequent data mining and web presentation.

**Conclusions:**

jMOTU efficiently and robustly identifies the molecular taxa present in survey datasets, and Taxonerator decorates the MOTU with putative identifications. jMOTU and Taxonerator are freely available from http://www.nematodes.org/.

## Introduction

The Linnaean project has already delivered species names for over a million taxa [1], but current estimates for the actual number of species on Earth range from 10 to 100 million [2], [3]. Molecular survey methods have been proposed as a practical solution to bridge the gulf between the desire and need to describe diversity and the number of hands and minds available to do the describing [4], [5], [6], [7]. These methods use the DNA sequence of a conserved gene or gene fragment and objective clustering rules to group the sequences into molecular operational taxonomic units (MOTU) [4]. We define MOTU as clusters of sequences (that act as representatives of the genomes from which they are derived) that are generated by an explicit algorithm. A dataset of sequences can be classified into MOTU at a number of different similarity cutoffs, the cutoff value acting as a parameter to the clustering algorithm. Initially applied in surveys of prokaryote diversity, these methods have revealed a hyperdiverse uncultured biosphere [8]. Similar surveys have been performed on microbial and meiobiotal eukaryotes, with the same summary findings: extant diversity may be orders of magnitude greater than described diversity [9], [10], [11]. The Barcode of Life project has proposed the use of such markers as useful species identifiers [12], and has embarked on a wide-ranging series of campaigns to collect ‘DNA barcodes’ from all animal, fungal and plant species [13], [14], [15].

As promoted by the Consortium for the Barcode of Life [12], a DNA barcode sequence is only valid if it derives from a vouchered specimen that has been identified to species. An unknown specimen can then be assigned to species if, and only if, its DNA barcode sequence matches that of a reference DNA barcode. This ‘Platonic’ approach has two (not insurmountable) problems [16], [17]: how close does a variant sequence have to be to the reference sequence to be assigned to a named taxon, and what does the system do with sequences (and thus specimens) that do not match to a known taxon? In prokaryotic DNA diversity surveys, the ‘Platonic’ approach has in general been sidelined because of the recognition that species-level description of Bacteria and Archaea lags far behind the true diversity of these groups. It is estimated that over 99% of bacteria are unculturable at present, and, as species descriptions generally require culture and phenotypic assay, over 99% of bacterial species-level taxa do not have a recognised name [8]. For prokaryotes therefore, analysis of sequence-based surveys of diversity has focussed on clustering of the individual sequences into MOTU using a sequence similarity cutoff derived from the known within-species diversity in the surveyed gene. These MOTU can then be analysed in the same way one would ‘true’ species. A similar approach can also be applied to non-prokaryote DNA barcode sequences, and, if the estimates of the taxonomy deficit for eukaryotic phyla are accurate, this approach may also be the only rational way of cataloguing eukaryotic diversity [16], [17].

As the size of DNA barcoding or ‘metagenetic’ surveys have grown from a few hundred dideoxy Sanger sequences to hundreds of thousands of Roche 454 pyrosequencing reads, the need for fast, accurate and robust algorithms for deriving MOTU from sequences has become critical [18], [19], [20], [21]. There are three main approaches to clustering, distinguished by how they treat distances between members of a cluster with respect to the distance cutoff (see [18] for a concise exploration of this). QIIME [21], ESPRIT [22] and Mothur [20] are high-performance workbenches for data analysis, but have significant dependencies. Here we present jMOTU and Taxonerator, programs for MOTU definition and taxonomic assignment, designed to be easy to install and use, and to be capable of analysing medium sized datasets. We demonstrate the utility of jMOTU in analysis of Sanger and Roche 454 nuclear small subunit ribosomal RNA and cytochrome oxidase I datasets at multiple distance cutoff values simultaneously. Taxonerator annotates MOTU with taxonomic information in order to aid assignment of MOTU and the sequences they include to traditional taxonomic identifiers.

## Results

### jMOTU algorithm

**Figure 1:**
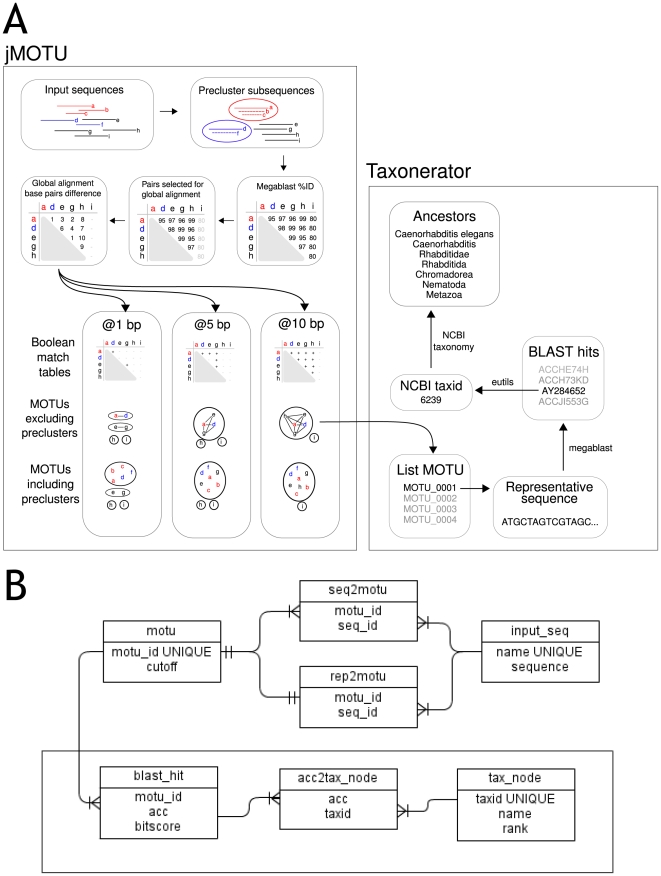
Outline of the jMOTU and Taxonerator pipeline. A: The jMOTU-Taxonerator workflow. The labelled grey boxes indicate the portions of the pipeline carried out by each program. Within jMOTU, input sequences are preclustered to remove exact subsequences, and representative sequences chosen for each precluster. Pairwise megablast scores are calculated for representative sequences, and exact distances between highly similar pairs are calculated using NW alignment. These exact distances are used to cluster the representative sequences into MOTU at various distance cutoff values. Within Taxonerator, each MOTU is processed separately. A representative sequence is chosen and used as the query in a megablast search of a preformatted database. The top 10 hits are extracted and their taxonomic hierarchy is stored for further analysis. B: The structure of the jMOTU (upper part) and Taxonerator (lower part, boxed)SQL database.

The workflow implemented in jMOTU is illustrated in Figure 1A. The input for jMOTU consists of one or more sequence files, and a set of parameters for MOTU definition (including one or more cutoff values). jMOTU does not use multiple sequence alignment, as this can introduce significant error [22], but derives distance data from pairwise NW alignments. To reduce the number of pairwise NW alignments required, jMOTU first removes redundancy in the input dataset by preclustering exact subsequence matches. It then chooses the pairs of preclusters to be NW aligned by performing all-against-all megablast [23] comparison of these preclustered sequences using a custom nucleotide similarity matrix, and filtering the megablast matches by comparing the score achieved to that expected for two sequences given the largest similarity cutoff requested. This approach combines the speed of local alignment using BLAST and the accuracy of global NW algorithms to achieve both high throughput and high accuracy. A matrix of absolute distances, ignoring differences due to insertion-deletion events (indels) and unresolved base calls, is computed for all analysed pairs from the NW alignments and used to define single linkage clusters for each cutoff value. Because single-linkage clustering is “greedy” and determinate, the resulting clusters are not affected by sequence order, and none of the members of a given cluster are closer than the cutoff to any member of any other cluster.

### jMOTU implementation

**Figure 2:**
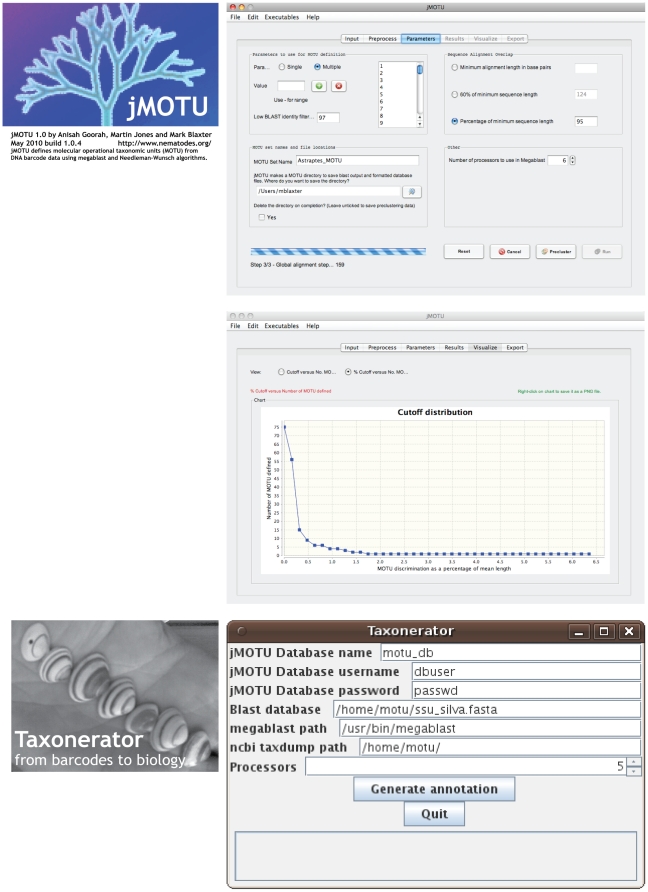
jMOTU and Taxonerator in action. Screeshots of (above) jMOTU's parameter pane, and display of MOTU numbers versus cutoff, and (below) Taxonerator's interactive data entry panel.

jMOTU is written in Java, and uses a graphical user interface to collect user input, display progress in analysis, and visualise outputs (Figure 2). jMOTU runs under Java 1.5 and 1.6. jMOTU requires that the BLAST suite of programs [23] is available on the user's system, and optionally uses the PostgreSQL relational database management system (http://www.postgresql.org/) to store the results of analysis.

The user should prepare DNA barcode sequences trimmed of linkers, adapters and other non-barcode data, and, preferably, trimmed for sequence quality. The user selects a FASTA-format sequence file containing multiple sequences, a directory of FASTA files or a NEXUS format file as input. Multiple sequence files can be loaded and analysed together. The sequences are loaded, and the distribution of sequence lengths is displayed. The user can choose to filter out short sequences at this stage. The user then defines the cutoff values at which MOTU should be defined (Figure 2). As distance data are calculated for the largest cutoff, there is little additional time cost for performing clustering at multiple cutoff values below the maximum. Indeed we would recommend this approach, as it is often unknown a priori what the optimal cutoff should be. Assessing a range of plausible cutoff values is a useful step in data exploration.

The user also sets values for the minimum overlap required between sequences (usually set to a high [> = 90%] value for barcode data where all sequences derive from the same PCR product) and a ‘gathering’ low megablast identity filter parameter (again usually set to a high [>95%] value) permitting the inclusion of BLAST matches that are just above the maximum cutoff value in the NW stage. The program then carries out preclustering and performs the all-against-all megablast to identify sequence pairs for NW alignment. The NW alignments are performed and a matrix of sequence distances built. MOTU are then inferred for each cutoff value. The clusterings can then be viewed (Figure 2), output as text files for further analysis, and output as an SQL file.

The SQL file can be used to populate a PostgreSQL database (Figure 1B). A relational database is well suited for the task of data exploration and can be used to collate analyses from multiple sequence datasets. We provide some example queries in the user guide.

### Taxonerator

Relating MOTU to classical taxonomy is an important step in integration of sequence-based surveys with classical knowledge of biology, life histories, feeding mode and distributions. Taxonerator adds taxonomic annotation to the PostgreSQL database generated by jMOTU (Figure 1, Figure 2). Taxonerator is written in Groovy, runs under Java 1.5 and 1.6, and requires a live internet connection, a preformatted database of taxonomically attributed reference sequences, a copy of the freely available text dump of the NCBI taxonomy hierarchy, and the BLAST suite of programs. We provide preformatted databases for cytochrome oxidase 1 (COXI; derived from the National Center for Biotechnology Information [NCBI] ENTREZ interface) and nuclear small subunit ribosomal RNA markers (nSSU; derived from the SILVA database [24]), and instructions for obtaining the NCBI taxonomy dump. Taxonerator queries the PostgreSQL database for the longest representative sequence for each MOTU, then identifies similar sequences in the reference database using megablast. The top ten matches are recorded, and their taxonomic assignment acquired by querying the NCBI EUtils web service. The full lineages (genus to kingdom) of the matches are obtained from the taxonomy dump. These annotations are then added to the PostgreSQL database (Figure 1B). Again, we provide example SQL queries that can be performed against the database to extract taxonomic information about MOTU.

### Use examples

Use of jMOTU and Taxonerator enables analyses of small to medium-sized DNA barcode datasets, delivering MOTU sets at multiple cutoff values with taxonomic attributions and numbers of sequences clustered in each MOTU. The time taken for the jMOTU process depends largely on the number of unique sequences present in the dataset being analysed. On a desktop computer (Mac G5) with 8 Gbyte of RAM, analysis of 47,000 initial sequences that were preclustered into 4,100 unique sequences took ∼4 hr. Taxonerator analysis is also dependent on the number of representative sequences that must be compared, but the above dataset analysed at cutoffs from 3 to 10 bases required 4 hr on the same workstation. If the starting data include many more unique sequences than this (>10,000) we recommend preclustering in batches, and combining preclustered sequences for a complete analysis (see the jMOTU user guide for details). Below we give two illustrative use examples.

### Use case 1: Astraptes ‘fulgerator’ cytochrome oxidase I barcode sequences

*Astraptes fulgerator* is a highly variable Neotropical skipper butterfly species that has been the focus of DNA barcoding research. In a landmark paper, Hebert and colleagues used COXI to investigate the taxonomic status of this species in Costa Rica [25]. Using DNA barcode, morphological and life history data, the single species *A. fulgerator* was proposed to contain at least ten distinct phylotypes, which were suggested to be ten species. While the species status of these groupings (and the taxonomic hypotheses used in their definition) have been criticised [26], the dataset remains a useful one for analysis. All 1088 COXI sequences from taxon *Astraptes* (NCBI taxonomy identifier [taxid] 283716) were downloaded from GenBank/EMBL/DDBJ. These sequences include 837 from txid 310673, the ‘*Astraptes fulgerator* complex’, 175 sequences from 11 species other than *A. fulgerator*, and 76 sequences from 8 taxa identified as just *Astraptes* sp.. From these assignments, and the claim in Hebert *et al.* [25] that *A. fulgerator* comprises at least 10 phylotypes, we expect between 19 and 29 taxa.

**Figure 3:**
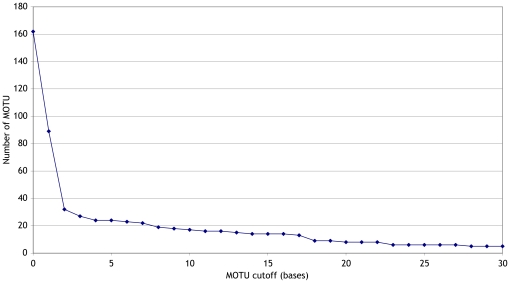
MOTU inferred in 1081 *Astraptes* cytochrome oxidase 1 sequences. MOTU were inferred using jMOTU at a range of cutoffs (x-axis). There were 162 0 bp MOTU, and 32 2 bp MOTU.

The sequences were analysed using jMOTU at cutoffs from 0 to 30 bases (Figure 3). Seven sequences less than 400 bases were excluded from the analyses, and the mean length of those remaining was 647 bases. There were 162 distinct sequences in the dataset. As the base cutoff for MOTU definition was increased there was an initial sharp fall in number of MOTU inferred, dropping to 32 MOTU at 2 bases difference (∼0.3% difference across 600 bases). This steep drop is what would be expected from analysis of data that include rare stochastic sequencing error and within-population variability. A 2% cutoff has been proposed as a general rule-of-thumb for taxon discrimination with COXI. At the 12 base cutoff (∼2% difference) there were 16 MOTU. Closer analysis of the taxon assignment of the sequences included in each MOTU shows that within the *A. fulgerator* complex, the sequence data alone do not support the proposed taxa (Table 1). Thus at the 12 base cutoff, while most of the sequences from *Astraptes* named species cluster as single MOTU (12bp_MOTU0002 to 12bp_MOTU0016), all of the 835 *A. fulgerator* complex sequences form one, 924-member MOTU (12bp_MOTU0001) along with 45 sequences assigned to *A. creteus*, 35 sequences in taxon *Astraptes sp.* Janzen02 and 9 sequences in *Astraptes sp.* hopfferiDHJ01. One sequence of three assigned to *A. fulgerator* with no subtaxon given forms a distinct singleton MOTU at the 12 base cutoff. Even at the 2 base cutoff, the *A. fulgerator* complex sequences lumped in 12bp_MOTU0001 do not robustly group by the names ascribed. Thus while most “SENNOV” and “YESENN” sequences are members of 2bp_MOTU0001 (along with sequences ascribed to LOHAMP and species *A. creteus*), other SENNOV and YESENN sequences form 2bp_MOTU0025, and a single SENNOV sequence forms 2bp_MOTU0032. Similarly, 2bp_MOTU0005 contains sequences from taxa “FABOV”, “INGCUP”, “HIHAMP” and “MYST”, but other sequences ascribed to these taxa form distinct MOTU.

**Table 1:** *Astraptes* MOTU.

12 base MOTU	2 base MOTU	*Astraptes* species or taxon assignment	Number of sequences
12bp_MOTU0001	2bp_MOTU0001	*creteus*	45
		LOHAMP	146
		SENNOV	137
		YESENN	262
	2bp_MOTU0002	CELT	44
	2bp_MOTU0004	Janzen02	35
	2bp_MOTU0005	FABOV	56
		HIHAMP	22
		INGCUP	94
		MYST	1
	2bp_MOTU0006	LONCHO	49
	2bp_MOTU0008	hopfferiDHJ01	9
	2bp_MOTU0012	MYST	6
	2bp_MOTU0019	INGCUP	1
	2bp_MOTU0022	HIHAMP	1
	2bp_MOTU0024	CELT	2
	2bp_MOTU0025	SENNOV	4
		YESENN	3
	2bp_MOTU0026	FABOV	1
	2bp_MOTU0029	ENTA	1
	2bp_MOTU0031	BYTTNER	4
	2bp_MOTU0032	SENNOV	1
12bp_MOTU0002	2bp_MOTU0003	egregiusDHJ02	5
	2bp_MOTU0018	egregiusDHJ01	3
12bp_MOTU0003	2bp_MOTU0007	*tucuti*	16
12bp_MOTU0004	2bp_MOTU0009	*enotrus*	20
12bp_MOTU0005	2bp_MOTU0010	*anaphus*	26
	2bp_MOTU0017	*anaphus*	13
12bp_MOTU0006	2bp_MOTU0011	*brevicauda*	16
12bp_MOTU0007	2bp_MOTU0013	*anaphus*	4
12bp_MOTU0008	2bp_MOTU0014	*apastus*	1
12bp_MOTU0009	2bp_MOTU0015	*talus*	10
12bp_MOTU0010	2bp_MOTU0016	janeiraDHJ02	9
12bp_MOTU0011	2bp_MOTU0020	cf creteus	10
12bp_MOTU0012	2bp_MOTU0021	*aulus*	5
12bp_MOTU0013	2bp_MOTU0023	*alardus*	13
12bp_MOTU0014	2bp_MOTU0027	*chiriquensis*	4
12bp_MOTU0015	2bp_MOTU0028	*fulgerator*	1
12bp_MOTU0016	2bp_MOTU0030	*phalaecus*	1

Thus objective clustering of the available COXI sequence data from the *A. fulgerator* complex does not offer independent support for the designation of distinct MOTU corresponding to those inferred by Hebert *et al.* [25], supporting the inference that the other characters used in the study (namely host food plant and caterpillar colour patterning) are those used to define these taxa, whose reality remains questionable [26].

### Use case 2: Roche 454 pyrosequencing analysis of meiofaunal diversity on a Scottish estuarine beach using nuclear small subunit ribosomal RNA

Roche 454 pyrosequencing can generate hundreds of thousands of sequences from target PCR amplicons in a single experiment. Roche 454 pyrosequencing data are known to be compromised by high systematic error rates associated in particular with difficulty in robustly measuring the length of homopolymeric nucleotide runs [27], [28], [29], [30]. These errors can result in significant inflation of taxon richness in deep-sequencing surveys [8], [31], [32], [33], and have prompted the development of software to correct Roche 454 sequences before or during clustering into OTU [29]. As the algorithm used by jMOTU ignores insertions and deletions (indels) in counting differences between sequences, jMOTU is relatively robust to homopolymer tract errors (which will appear as indels in a pairwise alignment).

We reanalysed the Roche 454 pyrosequencing dataset produced by Creer *et al.* [9] from an ecosystem study of the meiobiota (mainly Metazoa) at the low tide line on an estuarine beach at Prestwick in west Scotland. Eight size-sieved samples were taken from a low tide transect along Prestwick beach, and a ninth from Littlehampton, on the south coast of England, for comparison. From 18,004 to 51,952 sequences were generated per sample from bulk DNA extractions subjected to PCR amplification for the 5-prime end of nSSU (Table 2) [9]. In total, after filtering of short sequences (<200 bases), there were 292,397 nSSU sequences.

**Table 2:** Beach meiofaunal ecosystem survey.

dataset	Number of sequences	Number of unique sequences (0 base MOTU)	Number of 4 base MOTU
**Individual Samples**			
Prestwick 1	26120	3154	290
Prestwick 2	22995	2463	180
Prestwick 3	20127	2305	218
Prestwick 4	18004	2649	406
Prestwick 5	47144	4163	324
Prestwick 6	37285	5524	905
Prestwick 7	34140	5173	978
Prestwick 8	51952	8667	1470
Littlehampton	34630	4906	606
Totals	292397	39004	5377
**Pooled representative sequences**			
3 base MOTU representatives	6475	6094	3982

**Figure 4:**
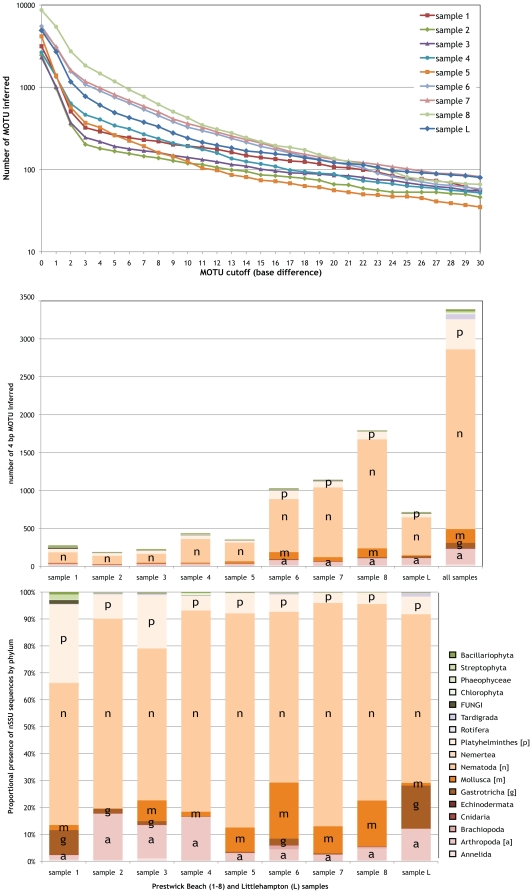
Analysis of community-sampled Roche 454 pyrosequencing barcode data. A 292,397-sequence dataset of nuclear small subunit ribosomal RNA gene fragments [9] was analysed using jMOTU and Taxonerator. A: For the 9 samples making up the dataset, the number of MOTU defined (y-axis; note log scale) at each base pair cutoff (x-axis) is shown. B: The numbers of 4 base MOTU inferred from each sample independently, and from the combined analysis of all representative sequences from each sample's 3 base MOTU. The stacked histogram bars indicate the assignment of these MOTU to animal phyla and other major taxa using Taxonerator (the key to colouration is in the lower right of the figure, and single-letter identifications for the major metazoan phyla are overlaid). C: Proportional presence in each sample of original sequences deriving from different animal phyla and other major taxa using Taxonerator annotations of the combined analysis 4 base MOTU (the key to colouration is in the lower right of the figure, and single-letter identifications for the major metazoan phyla are overlaid).

Memory usage by jMOTU is conditional on the number (*n*) of unique sequences being compared, as this defines the size of the similarity matrix (*n* by *n*). On the 8 Gbyte RAM computer being used for these analyses, the effective limit was ∼10,000 unique sequences. The full dataset exceeded this limit several fold, and thus we used a divide-and-conquer approach to analyse it. Each sample was analysed separately for MOTU cutoffs from 0 bases to 40 bases, yielding from 2305 to 8667 unique sequences (0 base MOTU) and from 180 to 1470 4 base MOTU (Table 2 and Figure 4 A). These MOTU were annotated using Taxonerator, based on the curated SILVA dataset of annotated eukaryotic nSSU genes [24], revealing that the majority of MOTU in each dataset derived from phylum Nematoda (plotted for 4 base MOTU in Figure 4B).

To analyse the complete dataset (all 9 samples) we used the jMOTU postgreSQL database to extract the representative sequences for all samples at the 3 base cutoff. These were then pooled, and second-tier jMOTU analysis was performed. The resulting MOTU were then annotated using Taxonerator and the SILVA eukaryotic nSSU database as before. As jMOTU and Taxonerator generate and store data at all requested cutoffs, it is possible to extract and further analyse at any chosen cutoff or taxonomic level. In Figure 4B we also show the analysis of the pooled dataset derived from the preclustered data. While eight of the nine samples came from an 800 m transect along the same beach, each sample contains a distinct subsampling of the overall diversity. Most of this unique diversity is present as MOTU with few sequences (the “rare biosphere”) but there are locally abundant MOTU. By cross-referencing from each input 3 base MOTU representative sequence to the number of original sequences that were present in the 3 base MOTU, we can sum the numbers of original sequences in each of the pooled analysis 4 base MOTU. Comparison between samples shows a consistent 50–70% representation by Nematoda (193,323 sequences generating 2367 4 base MOTU in the pooled samples), and high counts for Mollusca (25184 sequences, 182 4 base MOTU), Platyhelminthes (25000 sequences, 393 4 base MOTU) and Arthropoda (18740 sequences, 205 4 base MOTU). In the Littlehampton sample, and one of the Prestwick samples (sample 1), there were many sequences deriving from Gastrotricha (8660 sequences, 70 4 base MOTU). The method used for sample processing (size-selective sieving and flotation) did not exclude non-metazoan organisms, and thus we also identify 552 ‘protozoan’ (‘Protozoa’ is a paraphyletic assemblage; not figured in Figure 4), 23 fungal, and 65 viridiplantal 4 base MOTU. A small number of sequences formed 4 base MOTU with best matches to *Homo sapiens*, a probable contamination from the sampling team. Some nSSU remained unassigned (matching only ‘unidentified eukaryote’ sequences derived from other similar surveys).

At 4 base cutoff, there were a total of 3982 second-tier MOTU, of which 3328 were assigned to Metazoa. The rate of evolution of the nSSU is significantly less than that of COX1, species from the same genus may share identical nSSU sequences across the region sequenced. A 4 base cutoff as figured here thus probably corresponds to at best generic or subgeneric distinctness. While the absolute number of MOTU may be inflated due to PCR chimaeras, these will tend to be individually rare, and will generate low-member MOTU. About 30% of each individual sample's 3 base MOTU comprised single sequences, 80% of the second-tier 4 base MOTU derived from a single site, and overall ∼50% of second-tier 4 base MOTU had 2 or fewer sequences. To avoid counting PCR error as biological signal, one could accept as ‘real’ only MOTU that have at least a certain minimal number of members. However, the Taxonerator annotation of many of these low frequency sequences does not suggest chimaerism, and so a proportion does appear to derive from real, rare members of the meiofauna. These analyses are congruent with those performed by Creer *et al.* [9] using OCTOPUS (http://octupus.sourceforge.net/index.html) in the original publication.

### Availability

jMOTU and Taxonerator are available for download from http://www.nematodes.org/bioinformatics/jMOTU, including a virtual machine instance. The example datasets analysed in this paper are available on GenBank/EMBL/DDBJ; they are also available from the jMOTU website.

## Discussion

jMOTU has several properties that make it attractive for clustering of barcode datasets. It uses accurate pairwise distances, allows analysis of multiple cutoffs, is optimised to reduce runtime, and is insensitive to input sequence order. It is also easy to use.

jMOTU aims to use a distance metric that reflects the genuine genetic distance between sequences, as this is most likely to give clusterings that correspond to biological reality. To eliminate errors in distance estimation caused by alignment error, an exact distance is used, derived from an alignment calculated by the Needleman-Wunsch (NW) algorithm. Some existing clustering software calculates distances from multiple alignments that may be sub-optimal. Another potential source of error in distance estimations arises during sequencing. Pyrosequencing de-noising algorithms such as Pyronoise [29] aim to reduce this by within-dataset analysis, but are computationally costly. To minimise the contribution of sequencing error to the distance between two sequences, jMOTU only counts mismatches between nucleotides, ignoring positions where one sequence has a gap or an undetermined base. This avoids mistaking PCR or sequencing error for biological novelty, and, as MOTU clustering is usually performed to identify taxa at close taxonomic levels, effectively deals with the issue of how to code or score indels by assuming that most indels will be due to error in sequencing, and that real indels in closely related taxon groups will be rare. Since gapped positions are ignored, missing data is not taken into account, and thus the number of clusters estimated by jMOTU is conservative in the presence of missing data.

To accurately assess the distance between a pair of sequences, jMOTU uses the NW algorithm, which is guaranteed to find the best global alignment. Although the NW algorithm represents the gold standard for accuracy, it is computationally intensive compared to approximate alignment methods. jMOTU uses two strategies to minimise the number of global alignments that must be carried out. Preclustering reduces redundancy in the input sequence set and minimises the numbers of sequences that are involved in subsequent steps. Additionally, jMOTU avoids carrying out an all-against-all pairwise alignment by taking advantage of the fact that the clustering algorithm only requires exact distances between pairs of sequences that are relatively similar (i.e. those that will be clustered together under the most liberal cutoff). These pairs are identified using megablast, which, since it uses approximate alignments, is rapid even for large datasets.

jMOTU is designed to allow the user to explore patterns of clustering at different stringencies. Rather that choosing a single cutoff value to define the maximum distance between clustered sequences, jMOTU makes it easy to investigate the behaviour of the clustering algorithm using a range of cutoff values. It is able to do this efficiently by reusing the pairwise distance matrix to cluster at different cutoff values. The greedy clustering algorithm used by jMOTU ensures that clustering is not sensitive to input sequence order. A disadvantage to this algorithm is that new sequence data cannot currently be added to an existing dataset without re-analysing the entire dataset.

While currently unsuitable for single-pass analysis of very large datasets (involving more than ∼10^4^ unique sequences), we have demonstrated that by analysing subsets of the data individually, and then combining preclustered data in an overall analysis, jMOTU can effectively and efficiently deliver MOTU from these kinds of surveys.

Taxonerator represents an attempt to carry out first-pass taxonomic assignment of MOTU. For enviromental samples, we expect to encounter sequences that have no exact matches in known sequence databases, either due to sequencing error or true biological novelty. Rather than looking for exact matches, Taxonerator uses the most similar existing sequences to annotate a MOTU, which minimises the effect of sequencing error on taxonomic conclusions, and allows accurate taxonomic placement of true novel taxa. The user can specify a similarity cutoff for acceptance of annotation commensurate with the diversity expected in the experiment. Because Taxonerator stores information for multiple megablast hits, and for all nodes in each species' lineage, taxonomic annotation can be obtained for clusters at any taxonomic level. Importantly, for higher-level annotations (i.e. above the species level) the presence of sequencing errors will not affect the ability of Taxonerator to assign MOTU correctly, as the closest sequences will still be correctly identified. Chimaeras derived from PCR errors will tend to score poorly in terms of close matches to existing data, and thus are more likely to remain unannotated, or only annotated at high taxonomic levels. Additionally, the diversity of taxonomic annotation can be compared (e.g. across different sampling sites) at any taxonomic level. The graphical user interfaces for both programs enhance usability and assist the user in getting best practice analyses of their valuable data.
